# Biodegradation of Poly(ε-caprolactone): Microorganisms, Enzymes, and Mechanisms

**DOI:** 10.3390/ijms26125826

**Published:** 2025-06-18

**Authors:** Nikolay Krumov, Nikolina Atanasova, Ivanka Boyadzhieva, Kaloyan Petrov, Penka Petrova

**Affiliations:** 1 Institute of Microbiology, Bulgarian Academy of Sciences, 1113 Sofia, Bulgaria; krumov.1993@gmail.com (N.K.); nikolina@microbio.bas.bg (N.A.); petrovaim@abv.bg (I.B.); 2 Institute of Chemical Engineering, Bulgarian Academy of Sciences, 1113 Sofia, Bulgaria; kkpetrov@iche.bas.bg

**Keywords:** plastic, biodegradation, bacteria, fungi, esterase, lipase, cutinase

## Abstract

Poly(ε-caprolactone) (PCL) is a synthetic plastic known for its excellent physicochemical properties and a wide range of applications in packaging, coatings, foaming, and agriculture. In medicine, its versatility allows it to function as a scaffold for drug delivery, sutures, implants, tissue engineering, and 3D printing. In addition to its biocompatibility, PCL’s most notable characteristic is its biodegradability. However, this property is affected by temperature, microbial activity, and environmental conditions, which means PCL can sometimes remain in nature for long periods. This review shows that various types of microorganisms can efficiently degrade PCL, including different strains of *Pseudomonas* spp., *Streptomyces* spp., *Alcaligenes faecalis*, and fungi like *Aspergillus oryzae*, *Fusarium* spp., *Rhizopus delemar*, and *Thermomyces lanuginosus*. These microorganisms produce enzymes such as lipases, esterases, and cutinases that break down PCL into smaller molecules that act as substrates. The review also examines the phylogenetic diversity of organisms capable of biodegrading PCL, the biochemical pathways involved in this process, and specific aspects of the genetic framework responsible for the expression of the enzymes that facilitate degradation. Targeted research on microbial PCL biodegradation and its practical applications could significantly aid in reducing and managing plastic waste on a global ecological scale.

## 1. Introduction

**Figure 1:**
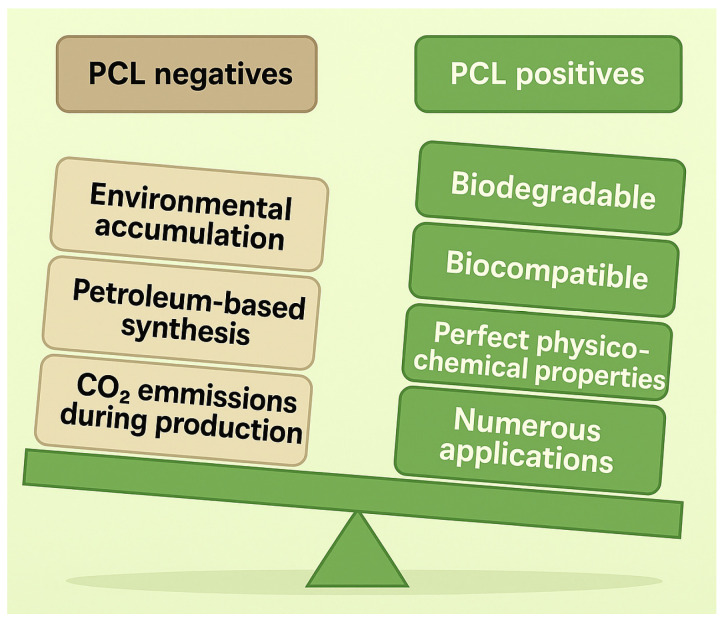
Pros and cons of using PCL.

Poly(ε-caprolactone) (PCL) is a synthetic biodegradable polyester known for its excellent physicochemical properties, thus finding wide applications in packaging, coatings, foaming, and agriculture [1,2]. Its chemical structure closely resembles that of polylactic acid (PLA) and polyhydroxyalkanoates (PHAs) [3,4]. However, unlike these biopolymers, its monomer, ε-caprolactone (ε-CL), is not biosynthesized by microorganisms but must be obtained through chemical synthesis [5]. As a petroleum-derived polymer, the production of PCL relies on non-renewable fossil resources, raising environmental concerns such as greenhouse gas emissions and the depletion of fossil carbon during production (Figure 1). Despite these drawbacks, PCL remains a more environmentally friendly alternative to non-biodegradable plastics such as polyethylene, polypropylene, and polyvinyl chloride, significantly contributing to global plastic pollution [6].

In 2022, the global market value for PCL reached USD 485.0 million, with projections suggesting it will increase to USD 1.1 billion by 2031, driven by a compound annual growth rate (CAGR) of 9.5% [7]. PCL offers several compelling advantages over other biodegradable polymers. For instance, PLA lacks impact resistance and exhibits poor thermal properties [4], whereas PCL is characterized by its rubber-like texture, high elasticity, and excellent mechanical strength. These properties render PCL highly suitable for applications that require flexibility and structural resilience. Additionally, PCL demonstrates superior solubility in various organic solvents, good compatibility in blends [7], and ease of processing, including techniques such as 3D printing and electrospinning [8,9]. Depending on the processing method, PCL shows slow degradation, which can benefit applications that demand long-term material stability, such as medical implants or agricultural films [10].

One of PCL’s most critical environmental attributes is its biodegradability. However, PHA, PCL, and their blends are not always biodegradable or may need conditions only available in industrial-scale composting facilities [9,10]. Efficient polycaprolactone (PCL) breakdown in natural environments relies on specific external factors, including temperature, salinity, microbial activity, and other ecological conditions. When these factors are suboptimal, the polymer’s persistent nature can lead to limited biodegradability and microplastic accumulation, posing long-term environmental risks [11,12].

The present review examines the potential of microorganisms as effective biodegraders of PCL, summarizing scientific data on the isolation and enzymatic characterization of various natural isolates from the past decade. The primary search engines used were Web of Science, Scopus, and Google Scholar, with a focus on peer-reviewed studies on PCL biodegradation, enzyme characterization, and biotechnological applications. Approximately two-thirds of the articles were published between 2020 and 2025. About 60% of the articles addressed bacterial strains with PCL-degrading activity, while approximately 30% focused on fungal species. Consequently, the extensive biodiversity of taxonomic groups and their enzymatic activities, genetic basis, and mechanisms of PCL degradation are detailed. Clarifying the specificity of various classes of microorganisms emphasizes some as particularly promising and indicates that PCL is a readily available substrate for them.

## 2. PCL Synthesis, Structure, Physicochemical Properties, and Applications

### 2.1. PCL Synthesis Methods

**Figure 2:**
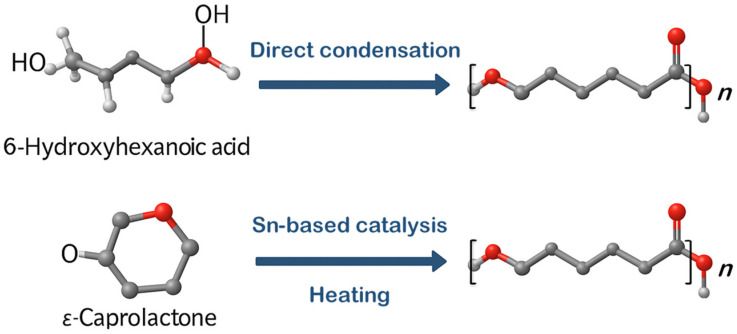
Two methods for synthesizing PCL: either through the direct condensation of 6-hydroxyhexanoic acid or by opening the ε-caprolactone ring in the presence of catalysts. Designation: *n*, variable number of monomers.

Poly(ε-caprolactone), or (1,7)-polyoxepan-2-one, can be synthesized from oil using various methods [13]. One approach involves the direct condensation of 6-hydroxyhexanoic acid (6-hydroxycaproic acid, 6-HCA), as illustrated in Figure 2 [11,14]. However, the most commonly used technique is the ring-opening polymerization (ROP) of ε-caprolactone (ε-CL) with catalysts (Figure 2). ROP is widely preferred in industrial settings for producing PCL due to its ability to yield polymers with higher molecular weights, narrower molecular weight distributions (lower polydispersity index), and better control over the polymerization process. The ring-opening polymerization (ROP) of ε-caprolactone typically utilizes one of three types of catalysts—anionic, cationic, or coordination–insertion—each producing polymers with distinct structural and molecular properties [15]. Anionic ROP relies on alkali metal salts such as potassium tert-butoxide, potassium isopropoxide, KH, phenyllithium, and lithium diisopropylamide as catalysts, where the ring-opening occurs specifically at the acyl–oxygen bond [13]. The active species in this process are alkoxide anions. A key limitation of anionic ROP is the occurrence of intramolecular transesterification, causing the formation of low-molecular-weight or cyclic polymers unless the reaction is halted beforehand. Nevertheless, this approach enables the controlled synthesis of high-molecular-weight polymers in polar solvents [16].

In cationic ROP, a positively charged intermediate is formed that is attacked by the monomer’s carbonyl oxygen, leading to ring opening via an SN2-like mechanism. This method is often challenging to control and generally results in polymers with low molecular weights or oligomers. Catalysts for this pathway include cationic aluminum complexes, trityl tetrafluoroborate (Ph_3_CBF_4_), and iron(III) chloride-based systems [17].

The coordination–insertion mechanism, or quasi-anionic ROP, involves coordinating the ε-caprolactone monomer to a metal center via its carbonyl oxygen. This reaction typically employs tin-based catalysts, including stannous (II) ethyl hexanoate, stannous (II) octoate, or stannous triflate [2,13]. The activated monomer then undergoes acyl–oxygen bond cleavage, allowing for the sequential insertion of monomers and the formation of the polymer chain. The end product can be chemically modified or blended with other materials to achieve the desired characteristics.

Sustainable PCL composites can be synthesized by initiating the ring-opening polymerization of ε-caprolactone using the surface hydroxyl groups of exfoliated oxidized biochar derived from hardwood waste biomass. This method enables neat, catalyst-assisted polymerization and produces composites with improved crystallinity, mechanical properties, and degradability compared to pure PCL [18].

Further, the possibility of producing or synthesizing ε-CL from renewable sources has also been investigated. Pyo et al. [5] developed a sustainable synthetic pathway for producing 6-HCA, adipic acid, and ε-CL by integrating biocatalysis and chemical catalysis. Bio-based 6-HCA was obtained from biomass containing glucose, fructose, or sucrose through the intermediate metabolite cyclohexanone, followed by the physicochemical conversion of 6-HCA to ε-CL using an ion exchange catalyst and a molecular sieve [5].

Alternative enzymatic methods include Baeyer–Villiger monooxygenases, such as cyclohexanone monooxygenase from *Acinetobacter calcoaceticus*, which catalyzes the oxidation of cyclohexanone to ε-CL in the presence of oxygen [19]. However, this enzyme is unstable and is inhibited by both the substrate and the product.

Another enzymatic method employs *Candida antarctica* lipase A, which facilitates the direct ring-opening oligomerization of ε-CL with up to 200 mM of cyclohexanol, yielding over 20 g/L of oligo-ε-CL [20]. However, this technique faces economic constraints due to the high cost of petroleum-derived cyclohexanol [5]. Previously, Dong et al. [21] utilized a lipase from Pseudomonas sp. to conduct ring-opening polymerization of lactones, achieving higher molecular weights and better monomer conversion compared to the condensation of the corresponding linear hydroxyesters and the copolymerization of ε-CL with lactide and cyclopentadecanolide [21].

### 2.2. PCL Structure and Physicochemical Properties

PCL is a linear, unbranched polymer in the polyester family, composed of up to 1000 repeating units of the cyclic ester caprolactone [22]. Its typical aliphatic structure contributes to its hydrophobic nature and solubility in organic solvents [23]. PCL exhibits various physical and mechanical properties depending on its chain length and molecular weight; higher-molecular-weight variants (e.g.,~40,000 Da) are rigid yet flexible. In comparison, lower-molecular-weight types like PCL-300 (10,000 Da) and PCL-150 (5000 Da) tend to be more brittle [24]. Being hydrophobic, the polymer is highly soluble at room temperature in organic solvents such as chloroform, toluene, and benzene. However, it does not dissolve well in acetone and acetonitrile, though solubility improves when heated to 50 °C [25]. It remains insoluble in water, alcohol, and petroleum ether [26].

PCL is a semi-crystalline polyester whose crystallinity decreases as molecular weight increases [27]. Its relatively low glass transition temperature (−60 °C) and melting range (58–64 °C) facilitate easy thermal processing but reduce stability at elevated temperatures—a limitation that can be addressed through blending or cross-linking [28,29]. PCL exhibits excellent thermoformability, making it widely used in 3D printing applications, where heating and melting are common fabrication steps [30]. It also demonstrates shape memory behavior [31,32].

PCL degrades slowly, typically over 2–3 years, and its high toughness is attributed to the presence of amorphous regions in its rubbery phase [13]. Table 1 compares the key physicochemical properties of PCL and other biodegradable polymers.

### 2.3. PCL Applications

**Figure 3:**
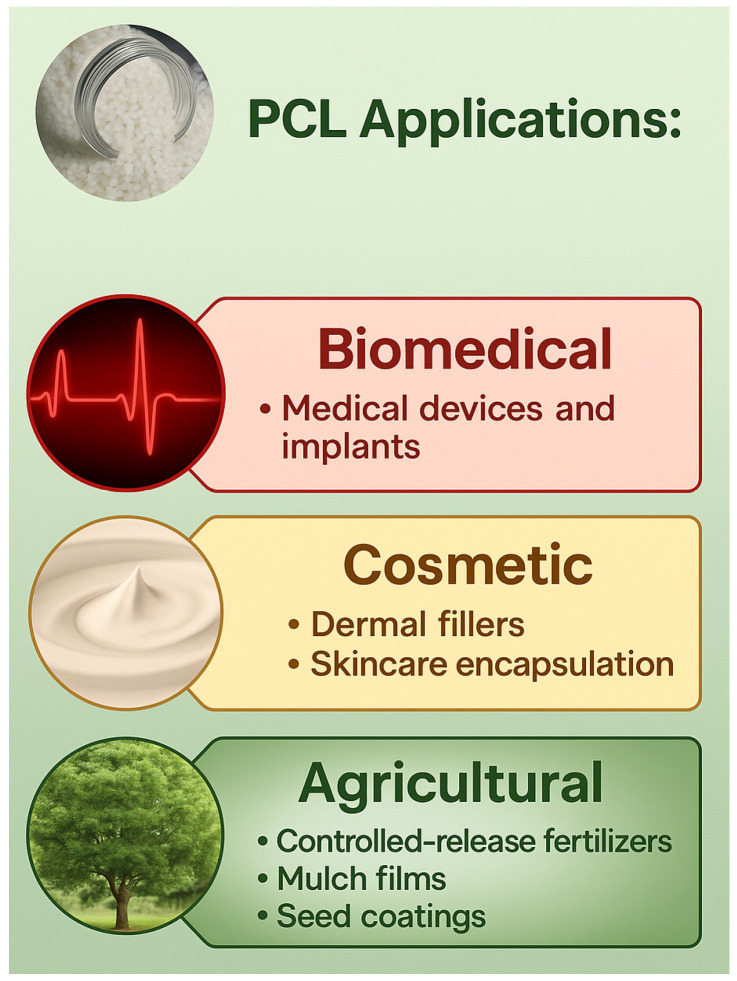
PCL applications.

Polycaprolactone (PCL) is widely valued across various industries for its distinctive thermoplastic behavior, biocompatibility, and hydrophobic nature. Its applications extend to the biomedical field, cosmetics, and agriculture (Figure 3), where it enhances product functionality, improves delivery mechanisms, and supports sustainable practices.

#### 2.3.1. Biomedical Applications

PCL has become a valuable material in the biomedical field due to its unique combination of biodegradability, ease of processing, and mechanical flexibility [33]. Given its advantageous rheological and viscoelastic behavior, the Food and Drug Administration of the USA (FDA) has approved PCL for use in long-term bioresorbable medical devices and implants, where it serves as a biodegradable alternative to traditional metallic components like screws and plates [34]. Its relatively low melting point enables thermo-responsive behaviors, making it ideal for advanced applications such as self-tightening sutures, biodegradable stents [35], and smart implants that can respond to body temperature and integrate seamlessly into surrounding tissues [36]. These characteristics contribute to developing less invasive surgical procedures and improved healing outcomes. PCL’s resistance to water further supports its use in drug delivery systems [7], bone and tissue scaffolds [37], and orthopedic fixation devices [38]. These applications leverage mechanical stability and gradual degradation over extended periods, which aligns with tissue regeneration timelines.

PCL’s excellent biocompatibility is a crucial property for various medical applications. When implanted, PCL does not elicit a strong immune or inflammatory response, allowing tissues in direct contact with it to heal wholly and swiftly [36,37]. However, the hydrophobic nature of PCL and its relatively slow degradation can restrict specific applications. As a result, researchers have concentrated on developing PCL-based blends and composites to address these limitations [7,9,12,30]. PCL composites frequently demonstrate improved hydrophilicity, mechanical strength, and tailored degradation rates, enhancing their biomedical potential [28]. For example, PCL/chitosan (CS) blends have demonstrated enhanced hydrophilicity and cytocompatibility. The double-porosity scaffolds created from PCL/CS facilitate cell adhesion and nutrient transport, making them ideal for bone and skin tissue engineering [7]. Lignin–PCL composites have also shown potential in neural tissue regeneration due to their antioxidant properties and ability to support Schwann cell proliferation [26]. Similarly, chitosan/PCL bio-inks have been optimized for lung tissue engineering through 3D printing, exhibiting excellent swelling, degradation, mechanical performance, and superior cell compatibility [37,38]. Blended PCL composites are also widely utilized in wound healing applications [39]. Their ability to support tissue regeneration, absorb exudate, and provide antibacterial properties renders them ideal for wound dressings. PCL/bacterial cellulose composites display high thermal stability and porosity, both advantageous for tissue repair [40]. Other blends, such as PCL/cellulose acetate/dextran with tetracycline, improve antibacterial activity while promoting blood clotting and cell proliferation [13]. Additional studies have indicated that incorporating Ag-containing nanoparticles into PCL scaffolds significantly enhances antimicrobial efficacy [41].

Due to its capacity for forming micro- and nanofibrous structures, PCL is a versatile platform for drug delivery. The large surface-area-to-volume ratio of electrospun PCL scaffolds enables high drug loading and controlled release [42,43].

#### 2.3.2. Cosmetic, Agricultural, and Environmental Applications

PCL has also gained attention in the cosmetics industry. Owing to its biocompatibility and slow resorption profile, it is employed as a dermal filler [44]. Additionally, PCL-based encapsulating agents are utilized in skincare formulations to enhance active ingredients’ targeted and sustained release, thereby improving product performance [45].

PCL’s agricultural applications utilize encapsulated controlled-release fertilizers that reduce nutrient loss and enhance crop efficiency. Furthermore, PCL is employed in the development of mulch films [30,45], seed coatings [46], and eco-composites [47], all of which help minimize plastic waste and improve soil health.

In addition to biomedical and agricultural applications, PCL-based blends serve as components in biosensors and optical sensors [13]. Furthermore, modified pectin/PCL blends are being researched as flexible, compostable films for biodegradable packaging, offering an eco-friendly alternative to traditional plastics [48].

## 3. Biodiversity of Microorganisms Capable of PCL Biodegradation

**Figure 4:**
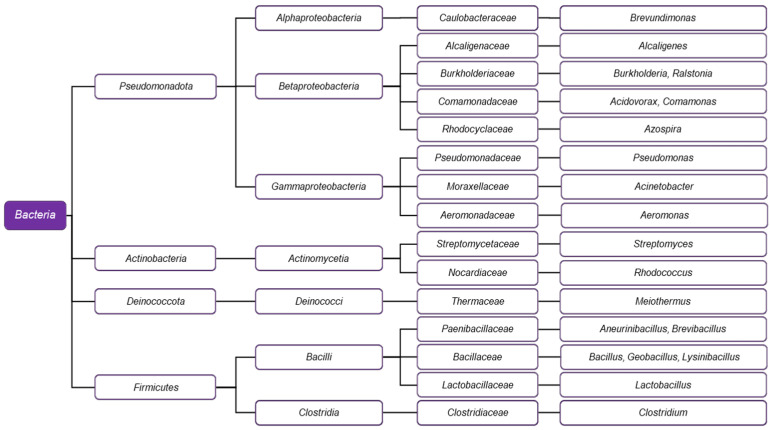
The phylogenetic diagram of bacterial genera capable of PCL degradation.

**Figure 5:**
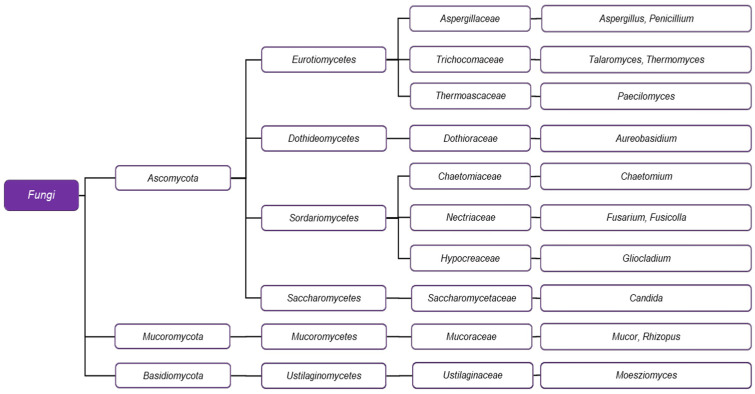
The phylogenetic scheme of fungal genera that are capable of PCL degradation.

Many microorganisms have been described in the scientific literature as capable of biodegrading PCL. Nearly all known species documented to perform such biochemical processes are either bacteria (Figure 4) or fungi (Figure 5), as archaea are rare [49].

### 3.1. PCL-Degrading Bacteria

Various classes within the *Bacteria* domain are recognized for their ability to degrade PCL, primarily represented by the phyla *Pseudomonadota*, *Actinobacteria*, *Deinococcota*, and *Firmicutes*.

#### 3.1.1. *Pseudomonadota* (Formerly *Proteobacteria*)

At least three major lineages of the formerly known phylum *Proteobacteria* can degrade PCL: *Alphaproteobacteria*, *Betaproteobacteria*, and *Gammaproteobacteria*. The *Alphaproteobacteria* are notable for *Brevundimonas* sp., a soil-isolated bacterium recognized for producing a powerful PCL depolymerase [49,50].

Among *Betaproteobacteria*, PCL-degrading species are found in the genera *Acidovorax*, *Alcaligenes*, *Burkholderia*, *Comamonas*, *Diaphorobacter*, and *Ralstonia* (order *Burkholderiales*), and in *Azospira* (order *Rhodocyclales*) (Figure 4). *Alcaligenes faecalis* has been found to hyperproduce the enzyme PCL depolymerase when cultured in a medium containing PCL [51]. *Burkholderia cepacia* has been shown to express a lipase utilized in the bioconversion of PCL within a two-phase system [52]. The genus *Ralstonia*, initially classified as *Pseudomonas*, is a close relative of *Burkholderia* and belongs to the same family, *Burkholderiaceae* [53]. Samples from a hot spring in Pakistan contained a thermophilic strain of *Ralstonia sp.* that effectively biodegrades PCL as a biofilm [54]. The *Comamonadaceae* family is notable for its genera *Acidovorax*, *Comamonas*, and *Diaphorobacter*, which include representatives that degrade PCL. Together with *Azospira*, another Betaproteobacterium, and the unrelated *Ignavibacterium* (*Fibrobacteres-Chlorobi-Bacteroidetes* superphylum) and *Frateuria* (*Gammaproteobacteria*), these microorganisms have been employed in a specialized aquaculture system, where they carry out a heterotrophic denitrification process using PCL as the sole carbon source [55].

Among *Gammaproteobacteria* degrading PLC are the representatives of the genera *Pseudomonas* and *Acinetobacter*, which belong to the order *Pseudomonadales*. The genus *Aeromonas* has been successfully employed in nitrification and denitrification processes, where PCL serves as a carbon source for growth, suggesting its potential application in wastewater treatment systems [56]. The effects of the *Pseudomonas* lipase enzyme have also been investigated. Scanning electron microscope (SEM) images of PCL–polyglycolide nanocomposites treated with this enzyme indicate visible degradation in the material’s structure [57]. In another study, a lipase from *Ps. chlororaphis* and *Streptomyces* strains grown on waste cooking oil was effective on PCL and polyhydroxyalkanoates [58]. *Ps. hydrolytica* has been found to express two different lipases, which have been successfully purified and characterized [59]. Compost isolates of *Pseudomonas pseudoalcaligenes*, currently considered a synonym of *P. oleovorans*, express cutinase when cultivated in a medium containing plant waste [60]. The genus *Acinetobacter*, a relative of *Pseudomonas*, is notable for a specific variety with biodegradative potential. Early studies with mixed cultures of *Acinetobacter calcoaceticus* var. *lwoffii* and the yeast *Cryptococcus laurentii* (syn. *Papiliotrema laurentii*) provide evidence that even polymeric chains with very high molecular weight are subjected to hydrolysis by the metabolic activity of the microorganisms [61]. Another member of the genus, *Ac. seifertii*, also demonstrates promising properties. Soil isolates, later identified as belonging to the species, exhibit a significant ability of their PCL depolymerase enzyme to degrade PCL effectively [62]. Furthermore, *Acinetobacter* has provided genes for 6-oxohexanoic acid (6-OHA) dehydrogenase, which have been utilized to develop a recombinant *Escherichia coli* strain with potential applications for eco-friendly poly-ε-caprolactone (PCL) upcycling [63].

The gammaproteobacterium *Aeromonas* attracts attention due to its ability to form biofilms on the surface of PCL and other plastic materials. A strain identified as *Aeromonas media* has been investigated in conjunction with a strain of *Rhodococcus quingshengii*. Both species showed the ability to degrade PCL with biofilm-forming properties and appeared resistant to a biocide introduced in cultured samples [64].

#### 3.1.2. Actinobacteria

In addition to *Proteobacteria*, another major phylum—*Actinobacteria*—also includes genera with similar metabolic capabilities. A prominent example is the genus *Streptomyces*, part of the order *Streptomycetales*. A thermophilic isolate of *Str. thermoviolaceus* subsp. *thermoviolaceus*, recovered from Taiwanese soils, degrades PCL via extracellular depolymerase, as shown by agar plate assays [65]. Researchers isolated two depolymerases from this microorganism, one displaying chitinase activity. The second key actinomycete genus is *Rhodococcus*, which belongs to the *Nocardiaceae* order. Two species, *R. erythropolis* and *R. opacus*, have provided essential insights into plastic degradation research. The genomes of these organisms have been sequenced and analyzed for the presence of genetic sequences responsible for PCL biodegradation, and several genetic determinants responsible for the process have been reported [66].

#### 3.1.3. *Firmicutes* and *Deinococcota*

Most members of the phylum *Firmicutes* that degrade PCL are limited to the class *Bacilli* and include the genera *Aneurinibacillus*, *Bacillus*, *Brevibacillus*, *Geobacillus*, *Lactobacillus*, and *Lysinibacillus*, with the genus *Clostridium* being the only exception. *Aneurinibacillus thermoaerophilus* and *Brevibacillus thermoruber*, both belonging to the family *Paenibacillaceae*, as well as the unrelated *Meiothermus cateniformans* (*Deinococcota*), are thermophilic bacterial species isolated from a plastic-contaminated hot spring in Bulgaria [67]. Through a series of morphological, microscopic, and enzymatic analyses, all three have demonstrated the ability to degrade PCL, with a strain of *Br. thermoruber* exhibiting the highest esterase activity in the collection [67]. *Bacillus* sp. strain NR4 degrades PCL alongside poly(butylene adipate-co-terephthalate), indicating that the genus may have a broader range of applications [68].

Another study shows that *B. pumilus* can also biodegrade PCL, which occurs naturally in the mangrove rhizosphere [69]. Moreover, a strain of *Bacillus* sp. has been utilized in developing bacterial fertilizers, where PCL serves as a material for creating an eco-friendly, biodegradable capsule [70]. *Geobacillus*, which belongs to the same *Bacillaceae* family, has been utilized as a source of lipase/esterase enzymes successfully engineered to degrade PCL with enhanced efficiency [71]. *Lysinibacillus* sp. also degraded PCL, as demonstrated by gel permeation chromatography [72].

PLC-degrading lactobacilli belong to the species *Levilactobacillus brevis* and *Lactiplantibacillus plantarum*. Three different lipases have been extracted from these species, as the lipase from *Lpl. plantarum* showed the highest activity [73]. Further investigation of this enzyme using an enzyme-embedded assay revealed significant physical changes to the plastic material resulting from the degradation process [74].

*Clostridium* is the most phylogenetically distinct example among the biodegraders within *Firmicutes*. However, at least two studies provide evidence that members of this genus exhibit this capability. Two strains of *Clostridium* sp. have been isolated from anaerobic sludge and evaluated for their biodegradative properties on PCL and two other plastic materials (polyhydroxybutyrate and the copolymer polyhydroxybutyrate-co-hydroxyvalerate). Following 16S rDNA analysis, the strains were identified as belonging to the species *C. acetobutylicum* [75]. In another study, *Clostridium* sp. isolated from aquaculture samples utilized PCL as a carbon source. The relatively high abundance of clostridia identified among the diverse array of microorganisms in the samples suggests that the genus may be essential in the biodegrading consortium [76].

#### 3.1.4. Putative PCL Degradation by Other Bacterial Species

Research on PCL biodegradation by bacterial species from phyla other than *Proteobacteria*, *Actinobacteria*, and *Firmicutes* is limited in the scientific literature. However, the taxonomic diversity of these microorganisms may be greater than currently recognized, considering that enzymes such as lipases and esterases, which are crucial to the process, are nearly ubiquitous among bacteria [77]. Members of their enzymatic family readily act upon PCL. Additionally, polyester, a plastic material chemically related to PCL, has been found to affect soil microbial communities by increasing the presence of bacteria from the phyla *Acidobacteria*, *Verrucomicrobia*, *Bacteroidetes*, *Nitrospirae*, and *Patescibacteria*, suggesting that these microorganisms may play a role in plastic degradation. It is essential to conduct in vitro cultivation of species from these taxa, along with experiments on the impact of PCL on their growth, which is critical for enhancing our understanding of the potential of bacteria to improve the exploitation of biodegradable plastics.

### 3.2. PCL-Degrading Fungi

PCL-degrading fungi show less taxonomic diversity in representative phyla than bacteria (Figure 5). Except for the basidiomycete *Moesziomyces antarcticus* and two members of *Mucoromycota*, *Mucor* and *Rhizopus*, the other studied genera—*Aspergillus*, *Aureobasidium*, *Gliocladium*, *Paecilomyces*, *Penicillium*, *Chaetomium*, *Talaromyces*, *Thermomyces*, *Fusarium*, *Fusicolla*, and *Candida*—are classified within *Ascomycota*. Both *Aspergillus* and *Penicillium* belong to the *Eurotiomycetes* class, and their biodegradative potential was recognized quite early, as demonstrated by the SEM of PCL, which was subjected to the action of *Asp. flavus* and *Pen. funiculosu*m [78].

Benedict et al. [79] conducted plastic degradation experiments involving *Asp. niger*, *Asp. fumigatus*, *Chaetomium globosum*, and *Fusarium* sp., demonstrating that all tested fungal strains produce low-molecular-weight material from PCL [79]. These fungi also degrade a blend of PCL and polyethylene as a consortium with two other species, *Gliocladium virens* and *Aureobasidium pullulans* [80]. Recent studies have highlighted *Chaetomium globosum*, which can fully degrade PCL in specific samples within 28 days [81].

Considering the final products of degradation, a thermotolerant strain of *Asp. fumigatus* has been discovered to produce a mixture of carboxylic acids from PCL film [82], similar to the purified lipase of *Asp. oryzae* [83]. Another *Aspergillus* strain sourced from a polluted seacoast in Korea has been studied for its ability to degrade PCL, with the authors noting that it, along with another ascomycete genus, *Talaromyces*, demonstrates higher activity than *Penicillium* [84]. Researchers have assessed the ability of *Aspergillus fumigatus* to degrade PCL in both soil and compost environments. These experiments also examined the properties of *Thermomyces lanuginosus*, another member of the *Eurotiomycetes* class, identifying it as a quick PCL microbial degrader [85]. It is noteworthy that although *Therm. lanuginosus* appears less frequently in plastic-related research, the enzymatic capabilities of this fungus warrant equal attention, given that a lipase from this species has been successfully utilized in vitro for the degradation of cross-linked PCL implants [86]. *Paecilomyces lilacinus*, also a representative of *Eurotiomycetes*, shows biodegradative capacity for both PCL and poly(3-hydroxybutyrate), as evidenced by a single study involving five different strains of filamentous fungi [87]. In contrast, the abilities of *Penicillium* species seem to be more evident in research on plastic degradation. Besides *Pen. funiculosum*, at least three other species from this genus have been studied for their effects on PCL, one being *Pen. oxalicum*. This fungus has been subjected to UV radiation mutagenesis, resulting in an effective biodegrading strain [88].

*Penicillium chrysogenum*, isolated from Russian soils, has been shown to degrade PCL, PLA, and polypropylene [89]. The most recently discovered fungi displaying similar activity are *Penicillium samsonianum* and *Fusicolla acetilerea*, both isolated from terrestrial samples in Korea [90]. *Fusarium*, a widely distributed soil fungus, is another genus of key importance for PCL biodegradation. In addition to being part of pioneering reports on fungal microorganisms demonstrating this ability, it has significantly contributed to understanding their enzymatic properties. *Fusarium* is a well-known plant pathogen, and as such, it relies on the activity of cutinase to lyse cutin [91], the outermost layer of many plant tissues. An analysis comparing a wild-type *F. moniliforme* and a knockout mutant of *F. solani* revealed that the cutinase functions as PCL-depolymerase [92]. Shortly after, a second depolymerase was isolated from *F. solani* and identified as a lipase distinct from the previously identified catalyst, indicating that biodegradation can be approached with different enzymatic strategies [93]. Moreover, researchers have successfully expressed a cutinase from *Fusarium solani* in the yeast *Yarrowia lipolytica* [94].

Another essential study targeting cutinase as a substrate showed that an extracellular protease can inactivate it [95]. More recent research has developed an enzymatic model for the hydrolysis of PCL through the activity of cutinase from *F. solani* and compared it to an accompanying model on the activity of a lipase from *Candida antarctica* [96], now reclassified as *Moesziomyces antarcticus*, thereby becoming the only currently known basidiomycete capable of biodegradation. The biodegradation process carried out by *M. antarcticus* has also been studied, with emphasis on the molecular weight of film-based PCL. While molecular weight does not significantly impact overall biodegradability, it does influence the size and depth of the hydrolytic pores and the time needed for the reaction to proceed [97].

Interestingly, in addition to its ability to biodegrade PCL, *M. antarcticus* has also demonstrated the ability to synthesize it, and the effects of two significant parameters on the process—temperature and organic media—have been evaluated [98].

Members of *Mucoromycotina*, the subphylum of *Mucoromycota*—including the common bread molds *Mucor* and *Rhizopus*—have also been reported to degrade PCL [99].

## 4. Mechanism of Microbial PCL Biodegradation

The microbial degradation of PCL proceeds through a sequence of three stages, each characterized by specific biochemical and physiological processes that convert the polymer into metabolic waste products while storing energy. These stages are as follows: (i) surface hydrolysis, where key enzymes cleave the bonds in the polymer backbone; (ii) absorption and digestion, involving the uptake and further assimilation of monomers and oligomers generated in the previous stage; and (iii) mineralization and excretion, during which the microorganism produces and releases small molecules into its environment.

### 4.1. Overview of Enzymes Involved in the Process of PCL Degradation

The key to microbial hydrolysis of PCL lies in the secretion of extracellular hydrolytic enzymes. Several lipases (EC 3.1.1.3) and some carboxylesterases (EC 3.1.1.1) are effective on this aliphatic polyester. Both groups of enzymes target the ester bonds in biological molecules, and depending on the specific enzyme, they participate in a broad range of biochemical processes. The precise distinction between lipases and esterases uses various criteria, including kinetic properties, structural features, substrate specificity, and hydrolysis mechanisms. Table 2 summarizes a widely accepted classification system for lipolytic enzymes, which organizes them into eight classes based on different structural and biochemical features.

### 4.2. Enzymes Involved in the Process of PCL Degradation by Bacteria

All esterases and lipases in bacteria share a characteristic α/β-hydrolase fold in their active sites, defining the catalytic strategy through which these enzymes carry out their functions. Most α/β-hydrolase fold proteins consist of six α-helices surrounding a central β-sheet composed of eight β-strands, nearly all of which are oriented parallel, with the only exception being strand β2, which runs antiparallel to the others. The β-sheet presents a distinctive half-barrel structure due to spatial twisting that positions the first and last strands at roughly a 90° angle to one another. Two of the α-helices flank one side of the structure, while the other four are located on the opposite side. Access to the active site for substrates is regulated by a set of secondary structures that form various lids, caps, or flaps. These features are particularly important for the lipase class (Class I), where a lid domain is present and plays a key role in interfacial activation.

**Figure 6:**
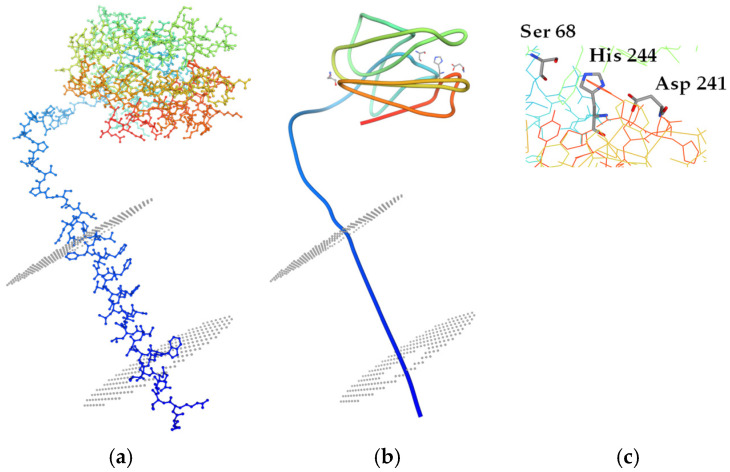
A 3D molecular model of the GDSL-type esterase/lipase family protein from *Brevibacillus thermoruber* strain 7, NCBI Reference Sequence WP_029099798.1. The sequence is deduced from genome mining of the whole genome shotgun sequence of *B. thermoruber* 423. The gene responsible, located in Scaffold 5, encodes a protein of 262 amino acids, which corresponds to the 28 kDa protein with PLA-degrading activity isolated by Atanasova et al. [67] from *Br. thermoruber* strain 7. (**a**) Ball and stick model presentation; (**b**) tube model; (**c**) catalytic triad.

In contrast to most other classes, Class II esterases (SGNH, or GDSL) lack the traditional α/β-hydrolase fold and instead exhibit a highly divergent α/β/α sandwich architecture (Figure 6). A notable difference is the β-sheet, which consists of five parallel strands (flavodoxin-like) in SGNH enzymes, contrasting with the eight-stranded structure typical of α/β-hydrolases. The oxyanion hole and the catalytic triad are present in GDSL esterases, operating through a similar mechanism. However, the amino acid sequence is generally conserved as Asp-His-Ser. GDSL esterases display distinct Nuc-Oxy and Acid–Base zones, forming an extensive network of hydrogen bonds in the active site that provides additional stability during substrate transition. The so-called SHLink, a two-bond interaction between residues adjacent to the catalytic nucleophile and base, connects these zones.

Class VIII esterases represent the most distinct category of bacterial lipolytic enzymes. Unlike the α/β-hydrolase fold, these enzymes have a β-lactamase-like fold, named for its similarities to β-lactamases. These esterases feature a two-domain architecture. The large domain consists of a β-sheet surrounded by α-helices. The β-sheet significantly differs from the α/β-hydrolase fold, as all its strands are arranged antiparallelly. The small domain contains four α-helices and two β-sheets with antiparallel strands. The binding site is located between the two domains. Another notable difference is the unusual catalytic triad, Ser–Lys–Tyr, where Ser acts as the acid component while Tyr is proposed to function as the base. Table 3 presents the main properties of bacterial enzymes exhibiting activity towards PCL.

**Figure 7:**
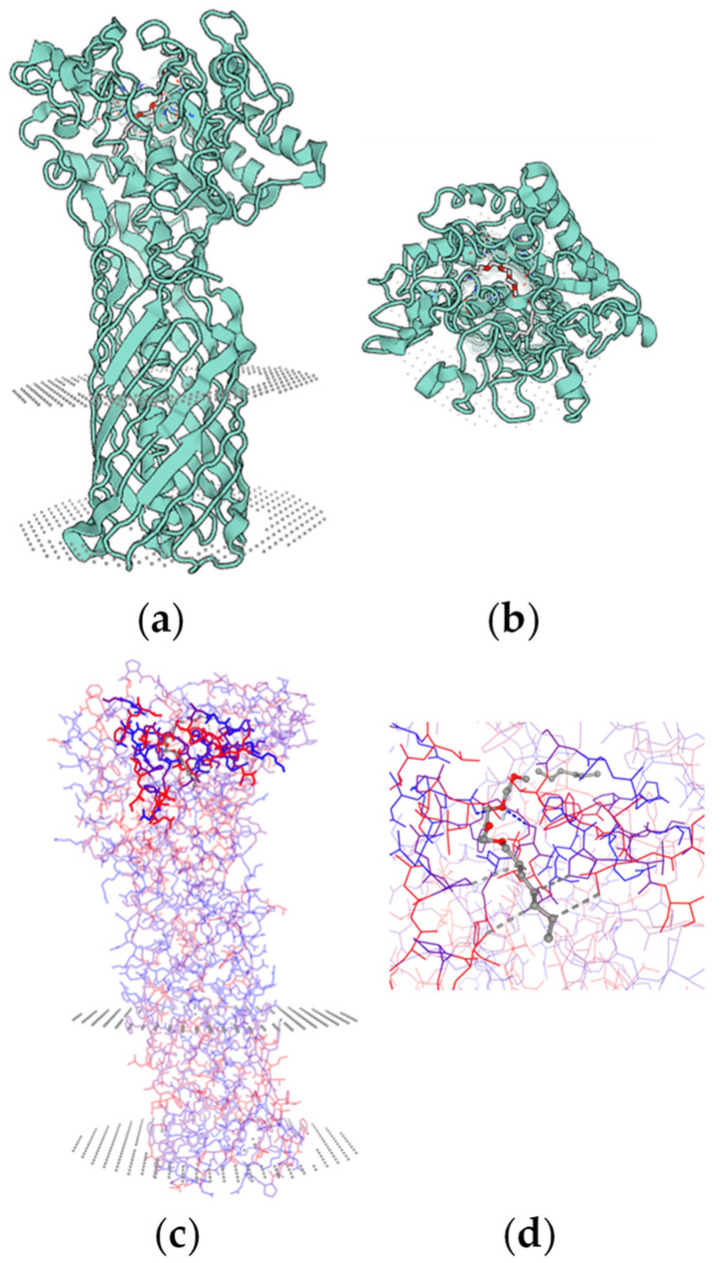
(**a**) Molecular model (3D) of the full-length carboxyl esterase EstA (E.C. 3.1.1.1) from *Pseudomonas aeruginosa* ATCC 15692, illustrating the protein’s contact with the outer cell membrane; (**b**) 3D view of the enzyme after a 90° horizontal rotation; (**c**) amino acid residues located in the hydrophobic pocket (illustrated in blue and red); (**d**) hydrophobic interactions with a model substrate (hydroxyethyl-oxy) tri(ethyl-oxy)octane). The SWISS-MODEL tool ‘Protein-Ligand Interaction Profiler’ predicts that eight amino acids are involved in hydrophobic interactions: L25, P198, I242, L293, and P300; S24 and N157, which form hydrogen bonds; and G102, which forms water bridges. The SWISS-MODEL Workspace https://swissmodel.expasy.org/ (accessed on 11 May 2025) was used to create the model and generate the prediction.

A common characteristic of all lipases is that they typically target water-insoluble molecules and require a hydrophobic interface (Figure 7). Hydrophobic interactions are significant, given that lipases possess a conserved hydrophobic region of amino acids in their active site (Figure 7c,d). Well-known lipase substrates include triglycerides, cholesterol, and specific vitamins, underscoring their essential role in fat metabolism.

In contrast, esterases generally act on smaller molecules that have higher water solubility [105,106]. Consequently, they often play a significant role in nucleic acid digestion, neurotransmitter inactivation, and the metabolism of xenobiotics, including drugs and toxic organic compounds. Regardless of the classification system, enzymes from the esterase and lipase classes have shown the ability to catalyze the breakdown of the polymeric PCL molecule into smaller products. This process begins when the secreted enzyme adsorbs to the material’s surface, followed by the formation of a temporary transition complex with the substrate. In the subsequent step, chain scission occurs, and the protein interacts with other molecular segments [107]. Experiments with polyethylene terephthalate, another member of the polyester family, indicate that biodegrading enzymes have a strong tendency for surface adsorption, making attempts to deliberately remove them from the plastic even more challenging [108].

### 4.3. Enzymes Involved in the Process of PCL Degradation by Fungi

The enzymes responsible for PCL degradation in fungi are lipases and cutinases. In addition to being subjects of scientific research, some are produced industrially (Table 4). One example is the lipase from *Candida rugosa*, although its PCL degradation level has been reported as low [89]. Similar results have been observed with the lipase produced by *Rhizopus del*emar. In contrast, the lipase from *Mucor miehei* has shown a sufficiently high degradation yield of the plastic, indicating that it may hold considerable significance for the practical application of the enzyme in this process [99].

**Figure 8:**
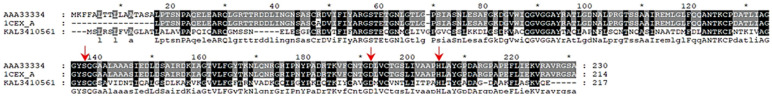
Comparison of amino acids in fungal cutinases (EC 3.1.1.74) using the free software GeneDoc v2.7. NCBI GenBank accession numbers and species: AAA33334, *Fusarium solani*; 1CEX_A, *F. vanettenii*; KAL3410561, *Asp. fumigatus*. Black shading indicates the conservation mode, red arrows highlight the amino acids involved in the active center, and asterisks indicate intermediate numbering of amino acids.

Cutinases (E.C. 3.1.1.74) are serine esterases originally associated with fungal invasion of plant cells by phytopathogenic fungi. In addition to their natural substrates, cutinases show activity toward a broad range of substrates, including PCL. Their structure is highly similar among *Fusarium* and *Aspergillus* species, and Figure 8 displays the conserved regions in the enzyme molecules.

### 4.4. Catalytic Mechanism of Enzymatic PCL Degradation

At least two experimentally supported models of the catalytic mechanism responsible for PCL hydrolysis propose a broadly comparable concept, though with some notable differences. The first model is based on the activity of an esterase from the thermophilic archaeon *Archaeoglobus fulgidus* (AfEST). This highly thermostable enzyme operates optimally at 80 °C and can synthesize polyester.

Three key amino acid residues, known as the catalytic triad—specifically Nucleophile–His–Acid—are responsible for the catalytic mechanism through which the α/β-hydrolase fold operates. The first and last residues may vary depending on the enzyme, while His is almost always conserved. The nucleophilic component is usually Ser, Cys, or Asp, and it is situated in the so-called nucleophilic elbow, representing a tight turn in the fold’s structure between αC and β5. On the other hand, the acidic residue is usually either Asp or Glu, and its function is to stabilize His in its protonated form. The mechanism involves two steps. First, the nucleophile attacks the substrate during acylation. Then, the process passes through two transition states before completing a deacylation reaction that releases the final product. In the AfEST model, aspartate (Asp) or glutamate (Glu), histidine (His), and serine (Ser) lie between the characteristic α/β hydrolase fold and the protein’s cap domain. The oxyanion hole, another crucial region, comprises three hydrophobic amino acids, two glycine (Gly) residues, and one alanine (Ala) residue. This region provides chemical stability by forming hydrogen bonds with its amide groups.

As the reaction progresses, the catalytic triad undergoes a cycle, starting with a nucleophilic attack by serine, which transfers a proton to histidine and continues with the formation of three successive intermediates: the first tetrahedral intermediate (INT-1), the acyl-enzyme intermediate (also known as enzyme-activated monomer, EAM), and the second tetrahedral intermediate (INT-2). The process concludes with the conversion to INT-2, which results in the recovery of the enzyme (RC), allowing the cycle to begin anew. The process includes four identifiable transition states: T1, T2, T3, and T4, each representing a specific step in the cycle. After the formation of INT-1 in the first step, another proton transfer occurs, cleaving a C-O bond in the substrate, leading to the release of 6-hydroxycaproic acid (6-HCA) from the PCL chain and the formation of EAM. EAM is subsequently subjected to deacylation by a nucleophilic attack from a water molecule, converting it to INT-2. In the final step, water establishes an equilibrium that drives the reaction toward releasing a second molecule, 6-HCA, and regenerating the enzyme (RC), accompanied by a decrease in Gibbs free energy, indicating that this step is exergonic. In conclusion, two 6-HCA molecules are cleaved from the PCL chain for each cycle turnover, representing the final product, which moves on to the next stage of biodegradation [109].

The second model centers on the thermophilic esterase MGS0156, identified through environmental metagenomic screening and capable of degrading various plastics, including PCL. Unlike AfEST, it operates optimally at a lower temperature (35–40 °C) and features a modified α/β hydrolase fold with a lid domain and a highly hydrophobic active site suited for binding PCL. As with AfEST, this model emphasizes the critical role of three catalytic amino acid residues in the reaction mechanism, characterized as a two-step process: triad-assisted nucleophilic attack and C-O bond cleavage. The model stresses that C-O bond cleavage is the rate-determining step, supported by a Molecular Mechanics Poisson–Boltzmann Surface Area Method (MM-PBSA). It indicates that different sets of six amino acids determine the binding strength of a PCL oligomer of a given size, noting that octamers demonstrate the highest binding energy. A valuable aspect of the study is the identification of two residues, His231 and Asp237, as targets for mutagenesis, providing implications for the bioengineering of enzymes derived from MGS0156 and their potential use as improved enzymatic tools in the biodegradation of PCL and other plastic materials [110]. Researchers have also noted other products resulting from the hydrolysis of PCL in addition to 6-HCA. An experiment comparing the abiotic and biotic degradation of PCL films, with the latter involving a mixed culture of compost microorganisms, shows that this process also leads to the release of various small molecules, namely caprolactone, a cyclic dimer, and a cyclic trimer, confirmed through a coupled analysis with gas chromatography and mass spectrometry (GC-MS). These products and 6-HCA are quickly assimilated and disappear entirely after two weeks in the biotic medium [111]. Interestingly, a GC-MS analysis of the degradation products in *Rhodococcus erythropolis* and *Rhodococcus opacus* shows a variety of other molecules, such as decanoic acid, undecanoic acid, dodecanoic acid, eicosane, pentacosane, and hexacosane, among others, but does not detect the presence of 6-HCA [112]. An earlier study examining the biodegradative potential of a thermotolerant strain of *Aspergillus* sp. identified the presence of succinic acid, butyric acid, valeric acid, and caproic acid after exposing plastic pellets to the activity of the microorganism at 50 °C. As anticipated, considering the chemical structure of its typical substrate, cutinase expressed by a strain of *Ps. pachastrellae*, isolated from coastal waters, results in 16-hydroxyhexadecanoic (Juniperic) acid, the main component of cutin, in addition to 6-HCA [113].

**Figure 9:**
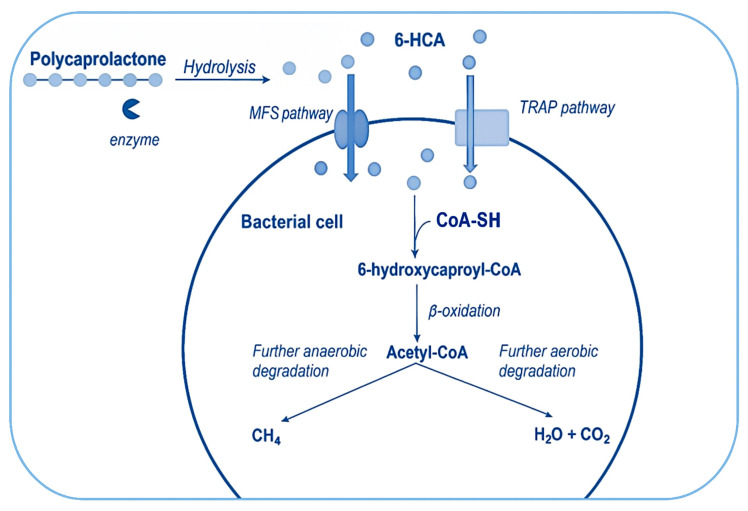
Proposed hypothetical model of the post-hydrolysis stage of PCL biodegradation. Designations: the thicker blue arrows indicate the possible transport pathways of 6-hydroxycaproic acid (6-HCA, blue circles) into the bacterial cell; the thin arrows indicate hypothetical biochemical conversions.

The post-hydrolysis stages of PCL biodegradation (Figure 9)—that is, the assimilation and further metabolization of lower-weight products followed by the excretion of waste molecules such as H_2_O and CO_2_ or, for some anaerobes, CH_4_—remain a largely unexplored area in bioplastic research.

Potential candidates for transporting 6-HCA across the cell membrane can be found among proteins from the major facilitator superfamily (MFS), representing an evolutionarily ancient lineage of molecular transport systems in bacteria. The MFS routinely imports carboxylic acids, including hydroxy acids like lactate, sugar acids such as gluconate, and various short-chain fatty acids [114,115]. Another possibility is transport via a member of the tripartite ATP-independent periplasmic transporter (TRAP) family, a common prokaryote-specific protein group known to engage in the secondary transport of a diverse array of carboxylates, including typical examples such as fumarate and malate—both of which are dicarboxylates—as well as pyruvate, lactate, L-glutamate, and others [116]. Since 6-HCA is chemically related to the aforementioned compounds as a carboxylic acid and exhibits a relatively simple chemical structure, it seems reasonable to focus on MFS and TRAP when investigating its transport mechanism. The fate of 6-HCA after it enters the bacterial cytoplasm is another critical area for potential experimental research.

Degradation through a catabolic pathway, such as beta-oxidation, is plausible since this process is the primary means of breaking down carboxylic acids of various chain lengths in many organisms, including bacteria [117,118]. Enzymes from the acyl-CoA synthase family can catalyze the formation of a thioester bond with CoA, allowing them to target a wide range of carboxylic acid molecules for degradation, resulting in products like palmitoyl-CoA [119], stearoyl-CoA, 3-hydroxybutyryl-CoA [120,121], isovaleryl-CoA [122], isobutyryl-CoA [123], and caproyl-CoA. The latter represents the activated form of caproic acid, which contains the same carbon atoms as 6-HCA, differing only by a single hydroxyl group.

Most current knowledge about the genetic factors governing PCL biodegradation comes from a pivotal set of experiments conducted with strains of *R. erythropolis* and *R. opacus*, two closely related aerobic species of actinobacteria. Zampolli et al. [112] have effectively determined the biodegradation capacity of these microorganisms by evaluating established hydrolytic zones (halos) on agar plates and conducting coupled GC-MS analysis of products extracted from bacterial cultures supplied with PCL. They have also measured high esterase activity through spectrophotometric tests, performing enzymatic reactions with five different organic substrates. The substrates include p-nitrophenyl acetate, p-nitrophenyl butyrate, p-nitrophenyl octanoate, p-nitrophenyl laurate, and p-nitrophenyl palmitate. The genome of *R. erythropolis* was subsequently sequenced via Illumina MiSeq v3, annotated, and then compared with the genome of *R. opacus* using the Rapid Annotations using Subsystems Technology (RAST) server.

Furthermore, the transcription levels of genes potentially involved in PCL biodegradation have been assayed using reverse transcription quantitative real-time PCR (RT-qPCR). An automated annotation of the genome of *R. erythropolis* reveals that it contains 6165 open reading frames (ORFs) and 72 RNA genes. Sequence-based and functional comparisons indicate that the two species share a substantial degree of identity, which the authors identify as a ‘common genomic core’. Interestingly, according to the RAST server, the closest neighbors of *R. erythropolis* are *R. jostii* and *Nocardia farcinica*. These findings are significant as they may indicate other microorganisms with potential biodegradation capabilities and suggest that genetic tools can be employed to identify such species. The RAST server also reveals that *R. erythropolis* and *R. opacus* share the same set of most populated functional categories, specifically amino acids and derivatives, carbohydrates, fatty acids, lipids, isoprenoids, cofactors, vitamins, prosthetic groups or pigments, and protein metabolism. The authors focus on genes involved in lipid metabolism, arguing that they are likely relevant to aliphatic polyester degradation, given the chemical similarities between the two groups of molecules. Further bioinformatic analyses have shown that this category includes a set of 10 and 9 genes encoding hypothetical proteins and lipases, which are potential objects of interest. Ultimately, RT-qPCR has revealed that three key genes from the selected group are expressed during the PCL biodegradation process: P133, which encodes a hypothetical protein; P253, which encodes a lipase; and P337, which encodes another hypothetical protein [112].

## 5. Conclusions and Prospects

PCL biodegradation offers a promising and sustainable approach to reducing plastic pollution while paving the way for eco-friendly material use. This review shows that various microorganisms, including bacteria, fungi, and archaea, can degrade PCL. The widespread presence of esterases and lipases in microbial communities indicates that the potential for biodegradation in nature may be significantly greater than documented. Exploring microbial diversity, particularly in extreme environments and under different ecological conditions, could reveal novel strains with enhanced biodegradative abilities.

A persistent challenge in this field is the limited understanding of the complete metabolic pathways involved in the assimilation and processing of PCL degradation products. While the enzymatic mechanisms of hydrolysis are relatively well characterized, the intracellular fate of intermediates such as 6-HCA remains largely unknown. Potential transport mechanisms, including the major facilitator superfamily (MFS) and tripartite ATP-independent periplasmic (TRAP) transport systems, are still hypothetical and have not been experimentally validated. Similarly, the specific roles of metabolic routes like β-oxidation in converting PCL-derived compounds into central metabolites require further clarification. Addressing these knowledge gaps is essential for optimizing microbial strains and enhancing biodegradation efficiency. Enzymatic models, such as those derived from *Archaeoglobus fulgidus* esterase and the metagenomic esterase MGS0156, provide mechanistic insight into substrate binding and catalysis, offering a valuable foundation for enzyme engineering. Advances in protein engineering, particularly directed evolution and rational design, could significantly enhance enzyme specificity, thermal stability, and catalytic performance, making them more suitable for large-scale bioplastic degradation and environmental remediation applications. However, translating laboratory success into large-scale biodegradation remains a considerable challenge. Environmental applications must consider microbial community dynamics, interactions with other pollutants, and abiotic factors such as temperature, pH, and nutrient availability. Utilizing complex microbial consortia instead of isolated strains may boost degradation rates while maintaining stability across varying environmental conditions.

Despite significant progress, several critical gaps remain in our understanding of microbial PCL biodegradation. Although many microorganisms and hydrolytic enzymes have been identified, most studies emphasize extracellular depolymerization under aerobic conditions, leaving anaerobic biodegradation pathways largely unexplored. The transport and intracellular metabolism of degradation products like 6-HCA are still poorly understood, particularly regarding how they are assimilated and integrated into central metabolic pathways. Furthermore, the genetic basis for the regulation and expression of PCL-degrading enzymes in various microbial taxa has yet to be fully characterized. Although genome and metagenome sequencing have revealed numerous putative enzymes, only a small percentage have undergone functional validation. Additionally, there is insufficient data on microbial consortia in complex environmental systems where PCL is present alongside other organic and inorganic contaminants.

In conclusion, a comprehensive approach that integrates microbiology, enzymology, synthetic biology, and environmental science is essential for advancing the field of PCL biodegradation. Future research should prioritize discovering novel microbial taxa, clarifying complete catabolic pathways, and developing engineered enzymes that function more effectively under environmentally relevant conditions. Addressing gaps in the existing literature will be critical for building a bridge toward scalable solutions in plastic waste management and improving the efficiency of PCL degradation in varied ecological settings.

## Figures and Tables

**Table 1:** Comparison of key physicochemical properties of PCL and other biodegradable polymers.

Property	PCL	PLA	PHA	Reference
Chemical structure	Aliphatic polyester	Aliphatic polyester	Polyhydroxyalkanoate	[2,4]
Melting temperature (Tm °C)	58–64 °C	130–180 °C	170–180 °C	[6,10]
Glass transition temperature (Tg, °C)	−60 °C	55–60 °C	−10 to 5 °C	[11]
Crystallinity (%)	45–70%	35–40%	30–80%	[4,11]
Degradation rate (in vivo/in vitro)	Slow (months–years)	Moderate (weeks–months)	Fast (depending on microbial strain)	[14]
Biocompatibility	Excellent	Moderate to high	Excellent	[1,2,11]
Solubility in organic solvents	High	Poor	Poor	[25,26]
Processability	Easy (low Tm, flexible)	Requires high temperature	Brittle, limited solubility	[28,29]
Shape memory effect	Present	Absent or weak	Absent	[31,32]
Mechanical behavior	Tough, flexible	Stiff, brittle	Variable, often brittle	[14]

**Table 2:** Classification system of esterases and lipases according to Arpigny and Jaeger [100], Holmquist [101], Denessiouk et al. [102], and Cea-Rama et al. [103].

Class	Type	Substrate Preference	Notable Features
I	Lipases	Long-chain triglycerides	α/β-Hydrolase fold, Interfacial activation, Lid domain
II	Esterases	Broad	Divergent, α/β/α sandwich; SGNH conserved sequence, GDSL motif near N-terminus (most)
III	Esterases	Short-chain esters	PAF-AH-like, α/β-hydrolase fold
IV	Esterases	Short-chain esters	HSL-like, α/β-hydrolase fold
V	Esterases	Variable	Dehalogenase and haloperoxidase-like enzymes in extremophiles
VI	Cutinases	Esters	Plant cuticle degradation
VII	Esterases	Acetylated compounds	Acetylcholinesterase-specific activity
VIII	Esterases	Broad	Distinct β-lactamase-like fold, untypical catalytic triad

**Table 3:** Enzymes that bacteria use to hydrolyze PCL.

Species/Strain	Source	Enzyme	Properties	Efficiency	Reference
*Brevibacillus thermoruber* strain 7	Marikostinovo Hot Spring, Bulgaria	Lipase	28 kDa, T range 45–65 °C, T_opt_ 55 °C, pH range 6–9, pH_opt_ 7–8, ↑Ca^2+^, ↓Mg^2+^, Co^2+^, K^+^, Na^2+^, Cu^2+^, Mn^2+^, Hg^2+^, Zn^2+^, Fe^3+^	Clear halos on 0.1% PCL-containing agar; deep surface damages on PCL after 1 week of incubation	[67]
*Brevundimonas* sp. MRL-AN1	Soil sample, Pakistan	PCL-depolymerase	63.49 kDa, T range 30–37 °C, pH range 6.0–8.0, ↓Fe^2+^ and Zn^2+^	80% of PCL film degraded in 10 days; prefers C_6_-C_10_ *p*-nitrophenyl acyl esters	[51]
*Alcaligenes faecalis* B273	Soil and activated sludge	PCL-depolymerase	T_opt_ 40 °C, pH_opt_ 7.0, K_m_ PCL = 0.29 mg/mL	Prefers C_10_ *p*-nitrophenyl acyl esters and tributyrin; does not cleave PHB	[52]
*Burkholderia cepacia* ST8	Soil and water, Malaysia	Lipase	↑Ca^2+^ ↓Cu^2+^ and Co^2+^	179 U/mL in medium with Tween 80	[53]
*Ralstonia* sp. MRL-TL	Hot spring	Esterase, serine hydrolase family	50 kDa, T_opt_ 50 °C, pH_opt_ 7.0, preferred substrate *p*-NP-caproate	50% of PCL film degraded in 10 days; it also degrades PLA, PES, PHB, and PHBV	[54]
*Pseudomonas pachastrellae*	Coastal seawater, Okinoshima Park, Japan	Cutinase	30 kDa, optimal at 0.51 M NaCl, operates via surface hydrolysis	Solid-state PCL-hydrolytic activity	[104]
*Pseudomonas* sp.	Sigma-Aldrich (Merck KGaA)	Lipase	T_opt_ 37° C in 0.05 M phosphate buffer solution (PBS) with pH 7	Visible degradation of the PCL matrix by the end of the first week	[58]
*Pseudomonas hydrolytica*	Lab-maintained strain, originally isolated from forest soil in China	PCLase I and PCLase II	PCLase I: 29,64 kDa; T 50 °C, pH 9; highly stable at pH 12 with 100% activity; ↑Mg^2+^, Ca^2+^, Fe^3+^, and Fe^2+^; ↓Cu^2+^ and Co^2+^ PCLase II: 33,2 kDa; T 40 °C; pH 10; ↑Mg^2+^ and Ca^2+^ at 1 Mm, ↑Co^2+^ and Cu^2+^, ↓Mg^2+^ and Ca^2+^ at 10 mM, ↓Fe^2+^	PCL I: degrades PCL (70% weight loss in 3 days), PBS, *p*NP ester, tributyrin, olive oil, and cutin PCL II: degrades PCL (75% weight loss in 8 days), PHB, PBS, *p*NP ester, tributyrin, and olive oil	[59]
*Pseudomonas pseudoalcaligenes*	Mixed-plant compost	Cutinase	32 kDa, consists of 302 amino acids, cutin-induced	Variable PCL degradation; clearing zones up to 65 mm	[61]
*Acinetobacter seifertii*	Soil samples	Esterase with PCL-depolymerase activity	30–40 °C	-	[62]
*Streptomyces thermoviolaceus* ssp. *thermoviolaceus*	Soil, Taiwan	Chitinase with PCL-depolymerase activity, PCL depolymerase	Chitinase with PCL-depolymerase activity: 35 kDa PCL depolymerase: 55 kDa	Both form bands in a polyacrylamide gel containing 0.1% PCL after incubation at 45 °C for 30 min	[66]
*Geobacillus* sp.	Lab-maintained strain, originally isolated from a Lithuanian oil well	Lipase/esterase, used for the development of engineered polyesterases: GD-95 RM and GDEst-lip in *E. coli*	GD-95 RM: LA 1400 U/mg, stable up to 85 °C GDEst-lip: 98 kDa, LA 600 U/mg; active between 5 and 90 °C, pH 6–12, both resistant to organic solvents, T_opt_ 50 °C	GD-95 RM: 264.0 mg and 280.7 mg of PCL_45,000_ and PCL_80,000_, 24 h at 30 °C GDEst-lip: 145.5 mg PCL_45,000_ and 134.0 mg PCL_80,000_, 24 h	[71]
*Levilactobacillus brevis*	Commercially obtained strain from IMTECH	Lipase	26 kDa, assayed at 37 °C, pH 7	10-day incubation; ~2 wt.% mass loss for 1 mg/mL lipase, increases to ~10 wt.% for 4–5 mg/mL	[73]
*Lactiplantibacillus plantarum*	Commercially obtained strain from IMTECH	Lipase	66 kDa, assayed at T 37 °C, pH 7; also at pH 8.1 + Tween 20 in an embedded approach	10-day incubation; ~10 wt % mass loss for 1 mg/mL lipase, increases to ~60 wt.% for 5 mg/mL; 73% mass loss in 8 days for 8% lipase-embedded PCL, crystallinity increases from 39% to 95%	[74]

**Table 4:** Enzymes involved in PCL hydrolysis by filamentous fungi.

Organism	Source	Enzyme	Properties	Efficiency	Reference
*Aspergillus oryzae*	Mucos Pharma ^1^	Lipase	Highest activity at Topt 37 °C, pH 7; retains stability at 55 °C	10% decrease in PCL Mw for 45 days of incubation	[73]
*Thermomyces lanuginosus*	Sigma Aldrich ^1^	Lipase	Topt: 37 °C, initial pH: 6.02, decreases during reaction	Mass loss over 5 days: ~54.8% at 0.1 mol% and ~17.3% at 3.0 mol% cross-linking. Corresponding degradation rates: 14.5%/day and 3.5%/day	[75,76]
*Paecilomyces lilacinus*	Soil, activated sludge	PCL depolymerase	T 30 °C; pH 3.5–4.5; expression inhibited by starch, glucose, and lactose	10% PCL degraded in 10 days	[86]
*Fusarium moniliforme*	University of Connecticut Culture Collection	Cutinase with PCL depolymerase activity	24 kDa, pH 9–10, induced by 16-hydroxy hexadecanoic acid	Zones of clearing on MM-PCL agar plates after 48 h	[82]
*Fusarium solani f. sp. pisi* 77–102	Institut fuÈr Genbiologische Forschung	Lipase/Cutinase ^2^	Lipase: pH 7.8, assayed at T 30 °C Cutinase: T range 20–70 °C, T_opt_ 30–40 °C, pH range 3–11, pH_opt_ 6–9	Lipase: clear MM/PCL/agar zones with Tween 80 or tributyrin; lipase/cutinase coexpression: 0.5 g PCL degraded in 144 h	[83,84]
*Candida rugosa*	Sigma-Aldrich ^1^	Lipase	T 40 °C, assayed at pH 7.7	PCL drops from 86,909 g/mol to 80,873 g/mol, and Mw remains at 1.48	[89]
*Mucor miehei*	Sigma-Aldrich ^1^	Lipase	T 40 °C, retains activity up to 60 °C, assayed at pH 7.7	74% PCL hydrolyzed 24 h, Mw of PCL drops from 86,909 g/mol to 24,011 g/mol, Mn/Mw increases to 2.39	[89]
*Rhizopus delemar*	Fluka ^1^	Lipase	T 40 °C, assayed at pH 7.7	PCL drops from 86,909 g/mol to 64,137 g/mol	[89]
*Moesziomyces antarcticus*	Beijing Cliscent Technology; Novozymes China Biotechnology ^1^	Lipase	33 kDa, 317 amino acids, T 45 °C, pH 7.2	87% PCL weight loss in 72 h via two-phase degradation: rapid (85%, 0–12 h) and slow (86.9%, 12–20 h), independent of PCL Mw	[86,87]

^1^ Commercially obtained enzyme; ^2^ lipase with PCL depolymerase activity; cutinase, coexpressed in *Y. lipolytica.*
